# Consistent Biofilm Formation by *Streptococcus pyogenes emm 1* Isolated From Patients With Necrotizing Soft Tissue Infections

**DOI:** 10.3389/fmicb.2022.822243

**Published:** 2022-02-18

**Authors:** Dag Harald Skutlaberg, Harald G. Wiker, Haima Mylvaganam, The INFECT Study Group, Anna Norrby-Teglund, Steinar Skrede

**Affiliations:** ^1^Department of Clinical Science, University of Bergen, Bergen, Norway; ^2^Department of Microbiology, Haukeland University Hospital, Bergen, Norway; ^3^Center for Infectious Medicine, Karolinska Institutet, Karolinska University Hospital, Huddinge, Sweden; ^4^Department of Medicine, Haukeland University Hospital, Bergen, Norway

**Keywords:** *Streptococcus pyogenes*, necrotizing soft tissue infection (NSTI), biofilms, M-protein, *emm1*

## Abstract

**Objectives:**

Biofilm formation has been demonstrated in muscle and soft tissue samples from patients with necrotizing soft tissue infection (NSTI) caused by *Streptococcus pyogenes*, but the clinical importance of this observation is not clear. Although M-protein has been shown to be important for *in vitro* biofilm formation in *S. pyogenes*, the evidence for an association between *emm* type and biofilm forming capacity is conflicting. Here we characterize the biofilm forming capacity in a collection of *S. pyogenes* isolates causing NSTI, and relate this to *emm* type of the isolates and clinical characteristics of the patients.

**Methods:**

Bacterial isolates and clinical data were obtained from NSTI patients enrolled in a multicenter prospective observational study. Biofilm forming capacity was determined using a microtiter plate assay.

**Results:**

Among 57 cases, the three most frequently encountered *emm* types were *emm1* (*n* = 22), *emm3* (*n* = 13), and *emm28* (*n* = 7). The distribution of biofilm forming capacity in *emm1* was qualitatively (narrow-ranged normal distribution) and quantitatively (21/22 isolates in the intermediate range) different from other *emm* types (wide ranged, multimodal distribution with 5/35 isolates in the same range as *emm1*). There were no significant associations between biofilm forming capacity and clinical characteristics of the patients.

**Conclusions:**

The biofilm forming capacity of *emm1* isolates was uniform and differed significantly from other *emm* types. The impact of biofilm formation in NSTI caused by *S. pyogenes* on clinical outcomes remains uncertain.

## Introduction

*Streptococcus pyogenes* causes a broad spectrum of disease manifestations ranging from mild, superficial infections to life-threatening invasive diseases, as well as post-streptococcal sequelae. This pathogen accounts for more than half a billion new cases and more than half a million deaths annually on a global scale (World Health Organization, 2005). Invasive *S. pyogenes* infections have a reported annual incidence of around 3 per 100,000 persons in the Scandinavian countries (Darenberg et al., 2007; Luca-Harari et al., 2008; Naseer et al., 2016). Necrotizing soft tissue infections (NSTIs) are acute, life-threatening conditions characterized by rapid and extensive destruction of the deep soft tissue (Sartelli et al., 2018). *S. pyogenes* is the most common pathogen in monomicrobial NSTIs (Skrede et al., 2020).

Biofilms are aggregates of microorganisms adhering to each other and to a surface and embedded within an extracellular matrix consisting of polysaccharides, proteins and nucleic acids. Microorganisms growing within a biofilm are largely protected against the host’s immune systems and the effect of antibiotics (Kumar et al., 2017).

*S. pyogenes* biofilm like structures have been demonstrated *in vitro* (Baldassarri et al., 2006; Lembke et al., 2006; Koller et al., 2010) as well as *in vivo* (Siemens et al., 2016). M protein, encoded by the *emm* gene, has an important role in *S. pyogenes* biofilm formation (Cho and Caparon, 2005; Courtney et al., 2009). However, there is conflicting evidence for a potential association between *emm* type and biofilm formation (Baldassarri et al., 2006; Koller et al., 2010; Ogawa et al., 2011; Wozniak et al., 2017). Other surface associated molecules known to be involved in *S. pyogenes* biofilm formation include pili (Manetti et al., 2007), streptococcal collagen like protein 1 (Scl1) (Oliver-Kozup et al., 2011) and hyaluronic acid capsule (Cho and Caparon, 2005). The clinical relevance of the *S. pyogenes* biofilm phenotype in mild infections of the upper respiratory tract and skin is recognized (Fiedler et al., 2015). In a previous study, we documented biofilm formation *in vivo* in NSTIs caused by *S. pyogenes* with biofilm demonstrated in tissue biopsies in over 30% of the cases (Siemens et al., 2016).

The aim of this study was to characterize *in vitro* biofilm forming capacity of *S. pyogenes* causing monomicrobial NSTI. We also wanted to explore possible associations between biofilm forming capacity and *emm* type of the isolates and clinical characteristics, including outcome, of the patients.

## Materials and Methods

### Study Population and Bacterial Isolates

Patients with confirmed monomicrobial NSTI caused by *S. pyogenes* prospectively enrolled in the INFECT project (ClinicalTrials.gov, NCT01790698), were included in this study. Demographic and clinical data were obtained from the INFECT trial database, described in detail elsewhere (Madsen et al., 2018).

Bacteria were isolated from sterile sites, including deep tissue or blood, and stored as frozen stock cultures at −80°C. Identification was performed using matrix-assisted laser desorption/ionization time-of-flight (MALDI-ToF) mass spectrometry. *emm*-typing and multi-locus sequence-typing (MLST) was done as described earlier (Bruun et al., 2020). Phylogenetic analysis was performed using CSI Phylogeny 1.4 available at Center for Genomic Epidemiology website (Kaas et al., 2014). The phylogenetic tree was edited in Geneious 9.1.7.^1^ Genomic sequences was retrieved from the European Nucleotide Archive (ENA database) under the BioProject PRJNA524111. One of the isolates (from case 2006) was used as a reference and included in every microtiter plate of the biofilm assay.

### Definitions

Necrotizing soft tissue infection is defined as necrotic or deliquescent soft tissue with widespread undermining of surrounding tissue as observed perioperative by the surgeon (Madsen et al., 2018).

Sepsis-related Organ Failure Assessment (SOFA) score is a tool to evaluate morbidity in critical ill patients based on respiratory-, circulatory-, renal-, coagulation-, hepatic-, and central nervous system function (Vincent et al., 1996).

### Generation of Biofilm Culture

Biofilms were formed according to a customization of a static biofilm culture model (Kwasny and Opperman, 2010). In short, portions of frozen stock cultures were first plated on sheep blood agar (SBA) and incubated overnight. A single colony from this first subculture was streaked on a fresh SBA plate and incubated for 18 ± 1.5 h. Colonies from second cultures were suspended in Tryptic Soy Broth containing 1% glucose (TSBG). The suspension was adjusted to a turbidity equivalent to a bacterial cell density of approximately 10^8^ colony forming units per ml (CFU/ml).

Wells of flat bottomed, 96 well, microtiter plates (Nunc A/S, Roskilde, Denmark, catalog no. 167008) were inoculated with 200 μl of a 10^–1^ dilution of this suspension. Inoculated microtiter plates were covered by a lid and placed at 4°C for 4 h (±20 min) in order to let the bacteria sediment without significant multiplication. The plates were then incubated at 37°C in ambient air for 18 h (±25 min).

Details of the microtiter plate lay out are shown in Supplementary Figure 1. In order to reduce evaporation from inoculated wells, all wells at the edge of the microtiter plate were filled with sterile TSBG. Six of these wells were used for blank correction of optical density (OD) measurements.

### Measurements of Biofilm Forming Capacity

At the end of incubation, the growth medium was removed by gentle aspiration. Bacteria loosely attached to the biofilms were removed by careful addition and subsequent aspiration of 300 μl Phosphate-buffered saline (pH 7.2). This rinse procedure was performed twice. Biofilms were heat fixed at 60°C for at least 90 min and thereafter stained using 0.06% crystal violet solution (50 μl) for 5 min at room temperature. Unbound stain was removed by gentle aspiration and the wells were rinsed five times as described above. Biofilms were then dried by leaving the plates without lid at room temperature for at least 60 min. Stain bound to biofilm was eluted by incubating the biofilms with 200 μl 30% acetic acid for 5 min. One hundred μl of thoroughly mixed eluate were transferred to a fresh microtiter plate and optical density was measured at a wavelength of 600 nm (OD_600_) using Synergy H1 microplate reader (BioTek, Winoosky, VT, United States). The mean blank-corrected OD_600_-value of eluate from six wells from each isolate served as a measure of biofilm forming capacity.

Viable bacterial density of the inocula was retrospectively verified by quantitative culture. If inoculum density was outside an acceptance range of 5.0 × 10^6^–1.5 × 10^7^ CFU/ml, the OD_600_-result was discarded and the actual isolate was re-tested.

### Statistical Methods

Normally distributed continuous variables are presented as mean [95% confidence interval (CI)] and compared using one-way ANOVA, while categorical variables are presented as numbers (percentages) and compared using Fisher’s exact test (two-tailed). Statistical significance was assessed after controlling the false discovery rate using the Benjamini-Hochberg procedure with a false discovery rate of 0.1. Quartiles were determined using the weighted average method. Between-run precision of the biofilm assay was determined using blank-corrected mean OD_600_-values of the reference isolate and expressed as coefficient of variation (CV). Statistical analyses were performed using IBM SPSS Statistics Version 24 (IBM Corp., Armonk, NY, United States). Power analyses were performed using G^∗^Power 3.1.9.2.

## Results

### Study Population and Bacterial Isolates

**FIGURE 1:**
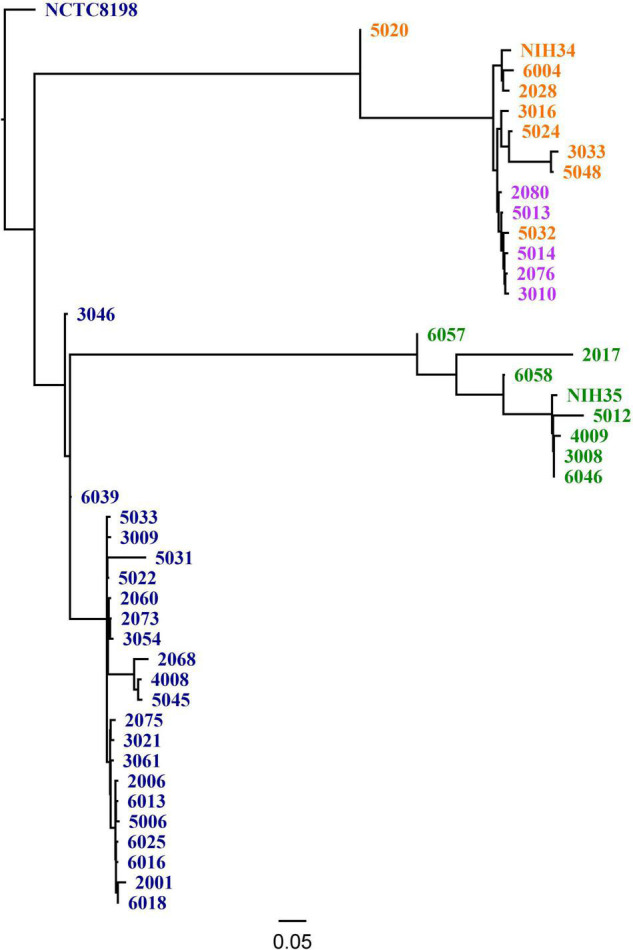
Single nucleotide polymorphism (SNP) based Phylogeny using CSI Phylogeny 1.4, including the three most common *emm* types in the study and reference strains [NCTC8198 (GenBank accession LN831034.1), NIH34 (GenBank accession AP023387.1) and NIH35 (GenBank accession AP023388.1)]. The color indicates *emm*- and ST type, including dark blue: *emm1* (ST 28); purple: *emm3* (ST 15); orange: *emm3* (ST 315) and green: *emm28* (ST 52).

One-hundred-and-fourteen (28%) of 409 patients included in the INFECT study had a monomicrobial infection with *S. pyogenes*. When this study was conducted, bacterial isolates were available from 59 cases. Of these, two cases were excluded because the density of bacterial inocula was repeatedly below the lower acceptance limit in the biofilm assay. Demographic and clinical data of the 57 included cases, are summarized in Table 1. The three most prevalent *emm* types, *emm1*, *emm3*, and *emm28*, comprised nearly three quarters of all isolates (Table 2). All *emm1* isolates belonged to sequence type (ST) 28 and all *emm*28 isolates belonged to ST 52. The *emm3* isolates were either ST 15 (*n* = 8) or ST 315 (*n* = 5), which differ from each other with a single base-pair in a single locus. With a few exceptions, the isolates within each *emm* type clustered close together (Figure 1).

**TABLE 1:** Biofilm forming capacity in relation to patient demographics, clinical variables and *emm* type.

Variables	Total	Biofilm forming capacity group^a^			*p*-value^b^
			
		Poor	Intermediate	Good	
Age in years, mean (95% CI), *n* = 57	58 (54–61)	54 (46–61)	59 (53–65)	58 (51–66)	0.49
Gender, n (%)					0.69
Female	30 (53)	7 (50)	17 (59)	6 (43)	
Male	27 (47)	7 (50)	12 (41)	8 (57)	
Comorbidities^c^, n (%)					1.00
Yes	31 (54)	7 (50)	16 (55)	8 (57)	
No	26 (46)	7 (50)	13 (45)	6 (43)	
Body part affected, n (%)					0.03
Upper extremities including thoracic involvement	26 (46)	5 (36)	18 (62)	3 (21)	
Lower extremities	19 (33)	7 (50)	4 (14)	8 (57)	
Head/neck, including intrathoracic space	8 (14)	2 (14)	4 (14)	2 (14)	
Abdomen and ano-genital area	4 (7)	0 (0)	3 (10)	1 (7)	
SOFA score day 1, mean (95% CI) (*n* = 55)^d^	9.4 (8.4–10.4)	9.0 (6.5–11.5)	9.2 (7.7–10.8)	10.1 (8.2–12.1)	0.70
Dead day 30, n (%)^e^					0.55
Yes	4 (7)	1 (7)	1 (4)	2 (14)	
No	52 (93)	13 (93)	27 (96)	12 (86)	
Amputation, n (%)^f^					0.90
Yes	9 (20)	3 (25)	4 (19)	2 (18)	
No	35 (80)	9 (75)	17 (81)	9 (82)	
*emm* type, n (%)^g^					0.000003*
*emm1*	22 (52)	0 (0)	21 (91)	1 (13)	
*emm3*	13 (31)	6 (55)	2 (9)	5 (63)	
*emm28*	7 (17)	5 (45)	0 (0)	2 (25)	

*^*a*^Poor: isolates with OD_600_-values below the first quartile. Intermediate: isolates with OD_600_-values above the first and below the third quartile.*

^*b*^An asterisk (*) after the *p*-value denotes a statistic significant result after correction for multiple comparison using the Benjamini-Hochberg procedure with a false discovery rate of 0.10.

*^*c*^Comorbidities: chronic obstructive pulmonary disease, cardiovascular disease, diabetes type I or II, chronic kidney disease, chronic liver disease, peripheral vascular disease, rheumatoid disease, chronic wound or other skin disease, varicella infection, active malignancy, metastatic carcinoma, hematologic cancer, HIV positive, other immunodeficiency.*

*^*d*^Two missing values for “SOFA-score day 1”: Poor biofilm forming capacity group: *n* = 13, Intermediate biofilm forming capacity group: *n* = 28 and Good biofilm forming capacity group: *n* = 14.*

*^*e*^Missing data for one case (*n* = 56).*

*^*f*^Only cases with limb affection (*n* = 44).*

*^*g*^Only the three most frequently encountered *emm* types are shown (*n* = 42).*

**TABLE 2:** Distribution of *emm* types.

***emm* type**	**Frequency (n)**	**Percent**	**Cumulative percent**
*emm1*	22	38.6	38.6
*emm3*	13	22.8	61.4
*emm28*	7	12.3	73.7
*emm12*	3	5.3	78.9
*emm22*	2	3.5	82.5
*emm77*	2	3.5	86.0
*emm87*	2	3.5	89.5
*emm89*	2	3.5	93.0
*emm4*	1	1.8	94.7
*emm25*	1	1.8	96.5
*emm58.5*	1	1.8	98.2
*emm180.1*	1	1.8	100.0
	57	100	

### Biofilm Forming Capacity

**FIGURE 2:**
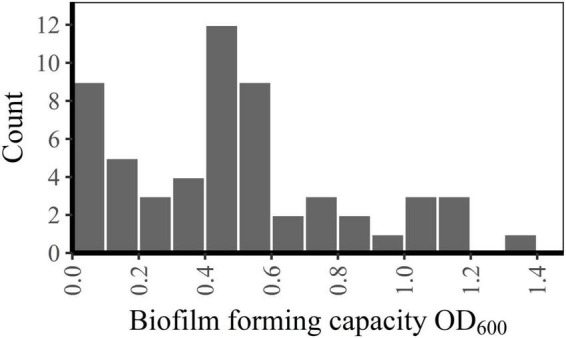
Frequency distribution of biofilm forming capacity.

**FIGURE 3:**
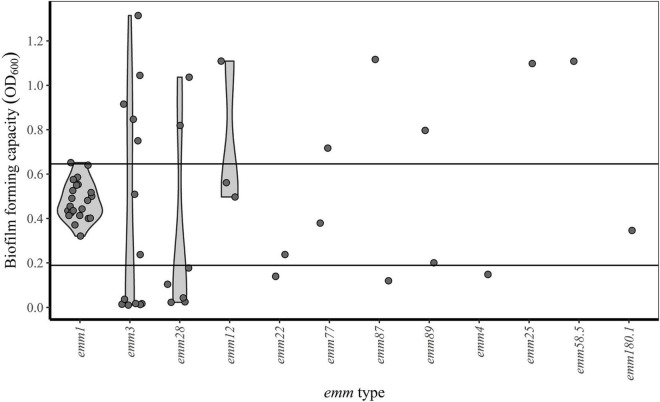
*emm* type and biofilm forming capacity. Horizontal lines indicate the first (OD_600_ = 0.19) and third (OD_600_ = 0.65) quartiles. Isolates with OD_600_-values below the first quartile, between the first and third quartile and above the third quartile are categorized as poor, intermediate and good biofilm formers, respectively.

Between-run CV for the biofilm forming capacity assay was 12.2% (data not shown). Biofilm forming capacity had a wide range (OD_600_-values from 0.01 to 1.31) and a multimodal distribution (Figure 2). However, all *emm1* isolates (*n* = 22) clustered closely within a narrow range (OD_600_-values from 0.32 to 0.65) and had a normally distributed biofilm forming capacity. Isolates other than *emm1* showed great variations in biofilm forming capacity, and only 14% (5/35) of these had biofilm forming capacity within the same range as the *emm1* isolates (Figure 3).

For comparative statistical analyses, isolates were classified according to their biofilm forming capacity: isolates with OD_600_-values below the first quartile (OD_600_ = 0.19), between the first and third quartile (OD_600_ = 0.65) and above the third quartile were categorized as poor, intermediate and good biofilm formers respectively (Table 1). The only *emm1* isolate categorized as a good biofilm former, clustered together with the other *emm1* isolates, but had an OD_600_ value slightly above the third quartile (Figure 3). While the *emm3* isolates were distributed across the whole range of biofilm forming capacity, the *emm28* isolates were either poor or good biofilm formers (Table 1 and Figure 3). For the remaining *emm* types there were too few observations to deduce a pattern (Figure 3).

**FIGURE 4:**
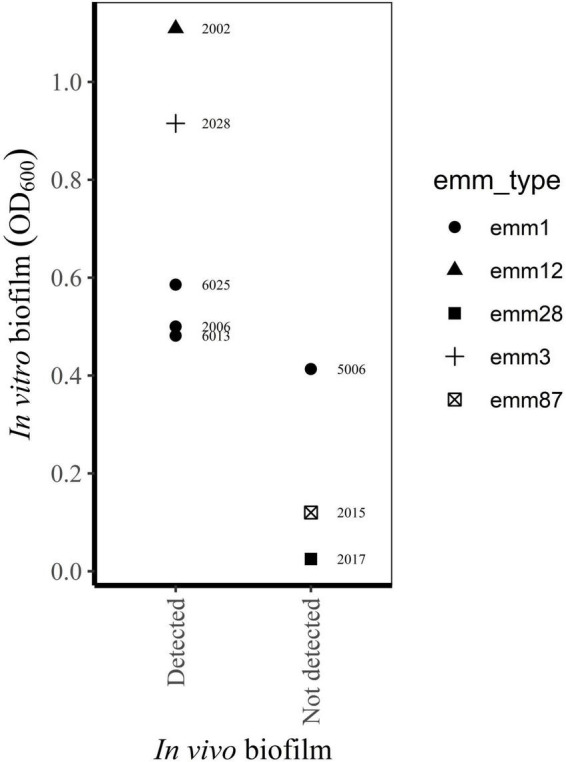
Relationship between biofilm *in vivo* (Siemens et al., 2016) and *in vitro* biofilm forming capacity of bacterial isolates from the same biopsies (this study).

Eight of the bacterial strains included in the present study are isolated from clinical biopsies thoroughly characterized with regard to *in vivo* biofilm formation by Siemens et al. (2016). The results from our *in vitro* model corresponded well to the presence of biofilm *in vivo*, as assessed by confocal laser scan microscopy and scanning electron microscopy (Figure 4).

## Discussion

The biofilm forming capacity of *emm1* isolates was uniform and differed significantly from other *emm* types. However, the associations between biofilm forming capacity and the different clinical variables were all non-significant.

The distribution of *emm* types in the present study fits well with results from previous prevalence reports of invasive streptococcal infections in the Scandinavian countries (Gherardi et al., 2018). Both the close relationship between *emm* type and sequence type (MLST) as well as the genomic stability among *emm1* isolates are in accordance with other reports (Enright et al., 2001; Barnett et al., 2018; Coppens et al., 2019; Li et al., 2020). Our finding that most *emm1* isolates are better biofilm formers than the majority of *emm28* isolates, is consistent with previous studies (Baldassarri et al., 2006; Koller et al., 2010; Ogawa et al., 2011). The only study among these with a sufficient number of *emm3* isolates, found a more homogenous distribution of biofilm forming capacity within this *emm* type compared to our data (Baldassarri et al., 2006). Other studies addressing the relationship between *emm* type and biofilm formation includes no or just a few *emm1*, *emm3*, and/or *emm28* isolates (Lembke et al., 2006; Thenmozhi et al., 2011; Wozniak et al., 2017), making a comparison to our results unreliable.

Lack of association between biofilm formation and clinical characteristics of patients with *S. pyogenes* NSTI is in accordance with earlier findings from our group (Siemens et al., 2016). Both studies use material from the INFECT project (Madsen et al., 2018), but whereas the previous study was based on assessment of biofilms in tissue biopsies (Siemens et al., 2016), the present study focuses on biofilm formation in an *in vitro* model. In addition, our study includes more cases (*n* = 57) than the previous study (*n* = 31). We are not aware of any other studies addressing the clinical significance of biofilm formation by *S. pyogenes* in NSTIs.

The main strengths of the present study are the prospective inclusion of cases, strict inclusion criteria and standardized collection of clinical data and bacterial isolates. As far as we know, this is the largest collection of *S. pyogenes* isolated from prospectively enrolled NSTI patients. Still the sample size is too small to conclude on a relationship between biofilm forming capacity and clinical characteristics of the patients, and this is a limitation in our study. *Post-hoc* power analyses of the different clinical variables, estimates powers of < 0.1, given a small effect size (Cohen’s *w* = 0.1), a significance level of *p* = 0.05 and the actual sample size (*n* = 57). Further, the categorization of bacterial isolates into biofilm forming groups may lead to misclassifications due to analytical inaccuracy. An examination of OD_600_-values close to the cut-off values, taking the between-run CV into account, reveals seven isolates (including four *emm1* and one *emm28* isolate) where analytical imprecision could have influenced categorization. However, these possible misclassifications will not affect the conclusion on an association between *emm* type and biofilm formation. Other limitations of our study is the *in vitro* design, disregarding the influence of host factors on biofilm formation, as well as the narrow spectrum of conditions used in the biofilm model. However, concordance between the *in vitro* biofilm forming capacity of selected isolates in this study and the presence of biofilm *in vivo* in corresponding biopsies (Siemens et al., 2016), indicates the usefulness of the *in vitro* model to predict biofilm formation in infected tissue (Figure 4).

Clonal relationship is a possible explanation of the uniform biofilm formation capacity demonstrated among *emm1* isolates in this study. In contrast, *emm3* isolates, display great variations in biofilm formation capacity despite close genetic relatedness within this *emm* type.

Some genetic variants are under positive selection in subsets of isolates (Lefebure and Stanhope, 2007; Carroll et al., 2011; Oliver-Kozup et al., 2011; Shea et al., 2011; Olsen et al., 2012), implying a beneficial effect in specific ecological niches. Importantly, genetic variants affecting the expression of virulence factors known to be involved in biofilm formation, may be unevenly distributed between isolates with the same *emm* type. Examples of this are surface associated structures like pili (Manetti et al., 2007; Koller et al., 2010), Streptococcal collagen like protein 1 (Scl1) (Oliver-Kozup et al., 2011) and hyaluronic acid capsule (Cho and Caparon, 2005; Shea et al., 2011), as well as the secreted streptococcal cysteine proteinase B (SpeB) (Carroll et al., 2011; Olsen et al., 2012).

Future research based on genomics may reveal the virulence factors’ contribution to the variations in biofilm forming capacity observed in the present study. However, genes may be variably expressed under different conditions or due to mutations in regulatory parts of the genome. Great differences between transcriptomes and proteomes at different time points of *S. pyogenes* biofilm formation, suggesting a substantial regulation by non-transcriptional mechanisms, underscores the importance of assessment at both the RNA and protein levels (Freiberg et al., 2016). Comparison of qualitative and quantitative expression of different genes, as well as detection of post-translational variants of proteins expressed during biofilm formation, could reveal components hitherto unknown to be important in streptococcal biofilm formation.

In summary, we have described the biofilm forming capacity in a collection of *S. pyogenes* isolated from patients with NSTI. We have demonstrated that invasive *emm1* isolates have a distinctive biofilm forming capacity compared to other invasive *emm* types, but were unable to detect associations of biofilm formation in NSTI to clinical outcomes.

## Members of the INFECT Study Group

Trond Bruun, Oddvar Oppegaard, Eivind Rath, Torbjørn Nedrebø, Ole Hyldegaard, Michael Nekludov, Mattias Svensson, Ylva Karlsson, Per Arnell, and Anshu Babbar. Affiliations for members of the Infect Study Group are detailed in the Supplementary Material.

## Data Availability Statement

The original contributions presented in the study are included in the article/Supplementary Material, further inquiries can be directed to the corresponding author.

## Ethics Statement

The studies involving human participants were reviewed and approved by the Regional Committee for Ethics in Medical Research (2012/2227) in Western Norway, the Danish Ethical Committee (1211709), the Swedish Ethical Committee (Dnr 930-12), and the Danish Data Protection Agency (30-0900). The patients/participants provided their written informed consent to participate in this study.

## Author Contributions

AN-T and SS: conceptualization. DS, SS, HW, and HM: methodology. DS: validation, formal analysis, investigation, writing original draft, and visualization. DS, SS, HW, HM, and AN-T: writing—review and editing. SS: project administration. AN-T, SS, and DS: funding acquisition. All authors contributed to the article and approved the submitted version.

## Conflict of Interest

The authors declare that the research was conducted in the absence of any commercial or financial relationships that could be construed as a potential conflict of interest.

## Publisher’s Note

All claims expressed in this article are solely those of the authors and do not necessarily represent those of their affiliated organizations, or those of the publisher, the editors and the reviewers. Any product that may be evaluated in this article, or claim that may be made by its manufacturer, is not guaranteed or endorsed by the publisher.

## Footnotes

1
https://www.geneious.com
