# Supplementary material for: Consistent Biofilm Formation by Streptococcus pyogenes emm 1 Isolated From Patients With Necrotizing Soft Tissue Infections
Source: Front Microbiol. 2022 Feb 18;13:822243. doi: 10.3389/fmicb.2022.822243 (PMC8895234; doi:10.3389/fmicb.2022.822243)
Supplement: Supplementary file 1 [file Data_Sheet_1.PDF]

Supplementary figure

Figure S1 Plate lay out for generation of biofilm culture

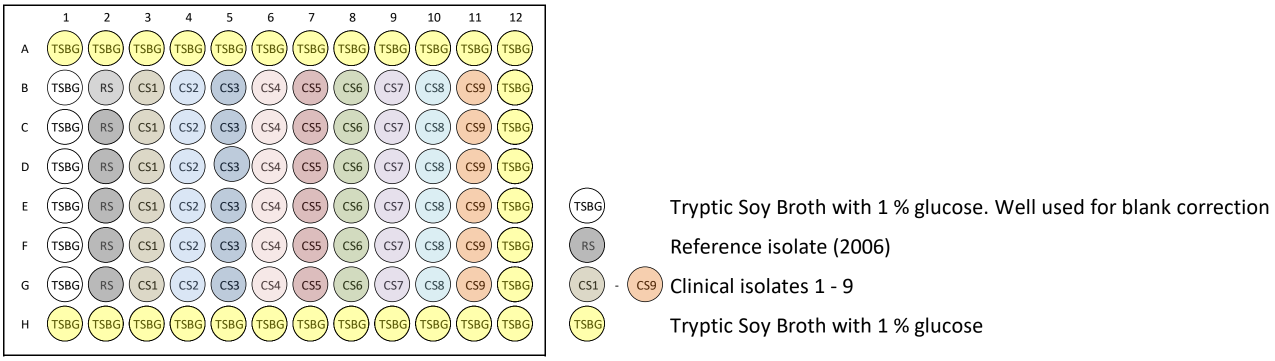

Supplementary table

Table S1 Strains used in the study

Case_ID	emm type
2001	*emm1*
2002	*emm12*
2006	*emm1*
2015	*emm87*
2017	*emm28*
2028	*emm3*
2056	*emm22*
2060	*emm1*
2068	*emm1*
2073	*emm1*
2075	*emm1*
2076	*emm3*
2080	*emm3*
3005	*emm89*
3008	*emm28*
3009	*emm1*
3010	*emm3*
3012	*emm77*
3016	*emm3*
3021	*emm1*
3033	*emm3*
3041	*emm12*
3043	*emm25*
3046	*emm1*
3054	*emm1*
3057	*emm89*
3061	*emm1*
4008	*emm1*

**Case_ID**	**emm type**
4009	*emm28*
5003	*emm77*
5006	*emm1*
5010	*emm22*
5012	*emm28*
5013	*emm3*
5014	*emm3*
5020	*emm3*
5022	*emm1*
5024	*emm3*
5031	*emm1*
5032	*emm3*
5033	*emm1*
5034	*emm87*
5042	*emm180.1*
5045	*emm1*
5048	*emm3*
6004	*emm3*
6013	*emm1*
6016	*emm1*
6018	*emm1*
6025	*emm1*
6026	*emm4*
6034	*emm58.5*
6039	*emm1*
6045	*emm12*
6046	*emm28*
6057	*emm28*
6058	*emm28*

INFECT study group: Author affiliation

Trond Bruun: Department of Medicine, Haukeland University Hospital, Bergen, Norway and Department of Clinical Science, University of Bergen, Norway
Oddvar Oppegaard: Department of Medicine, Haukeland University Hospital, Bergen, Norway
Eivind Rath: Department of Medicine, Haukeland University Hospital, Bergen, Norway
Torbjørn Nedrebø: Department of Anaesthesiology, Haraldsplass Deaconess Hospital, Bergen, Norway and Department of Clinical Science, University of Bergen, Norway
Ole Hyldegaard: Department of Anaesthesia, Centre of Head and Orthopaedics, Copenhagen University Hospital, Rigshospitalet, Copenhagen, Denmark
Michael Nekludov: Perioperative Medicine and Intensive Care Function, Karolinska University Hospital, Stockholm, Sweden.
Mattias Svensson: Department of Medicine, Center for Infectious Medicine, Karolinska Institutet, Huddinge, Sweden
Ylva Karlsson: Department of Anaesthesia and Intensive Care, Blekinge County Hospital, Karlskrona, Sweden
Per Arnell: Department of Anaesthesia and Intensive Care, Sahlgrenska University Hospital, Gothenburg, Sweden.
Anshu Babbar: Microbial Interactions and Processes, Helmholtz Centre for Infection Research, Braunschweig, Germany
